# Experience of using anti-CD 20 therapies in multiple sclerosis patients in Kenya

**DOI:** 10.3389/fneur.2025.1681527

**Published:** 2026-01-26

**Authors:** Nyambane Eunice, Tejal Patel, Jacqueline Mavuti, Juzar Hooker, Dilraj Sokhi

**Affiliations:** Aga Khan University Hospital Nairobi, Nairobi, Kenya

**Keywords:** multiple sclerosis, anti-CD 20, B-cell therapy, rituximab, ocrelizumab, Kenya

## Abstract

**Objective:**

The objective of the study was to evaluate the safety and efficacy of ocrelizumab (OCR) and rituximab (RTX) in multiple sclerosis.

**Methods:**

This was a retrospective single-centre study. Ocrelizumab- and rituximab-treated patients were identified through the multiple sclerosis (MS) registry maintained at Aga Khan University Hospital Nairobi (AKUHN), Kenya. Adult patients aged 18–65 years old who fulfilled the McDonald 2017 diagnosis criteria and received treatment with either rituximab or ocrelizumab between January 2016 and June 2025 were retrospectively evaluated. Data collected at baseline included age, gender, first symptoms, disease duration since onset, MS phenotype, treatment duration, previous therapies, reasons for switching to anti-CD 20 (cluster of differentiation) therapy, date of start of anti-CD 20 therapy, and adverse events. Disease activity was evaluated both clinically and through magnetic resonance imaging (MRI).

**Results:**

A total of 67 patients (male:female, 14:53) received anti-CD 20 therapy, with the majority having relapsing–remitting MS (RRMS) (5277.6%), while the rest had progressive MS. Patients were treated with either ocrelizumab 600 mg or rituximab 1,000 mg administered intravenously (IV) every 6 months. After 1 year, the cumulative relapse rate dropped, with the number of patients having clinical relapse events reduced from 48 to 7. Overall, 40 patients had stable MRI findings, 7 had new MRI lesions, and 20 did not have follow-up scans. No infusion-related adverse events or life-threatening infections were reported with the administration of anti-CD 20 therapy, and no case of malignancy or progressive multifocal encephalopathy was detected.

**Conclusion:**

This retrospective, single-centre study provides real-world data on B-cell-depleting therapies in an African MS cohort. Ocrelizumab and rituximab appear to be safe, well-tolerated, and effective therapeutic options for people living with MS.

## Introduction

Multiple sclerosis (MS) is a chronic, immune-mediated demyelinating disease of the central nervous system with both inflammatory and neurodegenerative characteristics (1). MS has diverse clinical phenotypes, namely relapsing–remitting MS (RRMS), progressive MS, and clinically isolated syndromes (CIS). RRMS follows an unpredictable chronic course characterised by alternating periods of remission and disease activation. Progressive MS is characterised by a gradual worsening of symptoms over time, which may or may not be accompanied by inflammatory activity. These phenotypes have varying neuroradiological findings and differing responses to treatment (2, 3).

Early use of high-efficacy therapy rather than escalation has been associated with better outcomes in MS (4). B-cell-targeted therapy has emerged as an efficacious option for the treatment of MS due to extended dosing intervals (5). While rituximab is not officially approved by the Food and Drug Administration (FDA) or European Medicines Agency (EMA) for use in MS, it has been used as off-label treatment in several countries and was recently added by the World Health Organization to its essential medicine list for MS (6). Rituximab has been available at the Aga Khan University Hospital Nairobi (AKUHN) since 2016 and has been used for the treatment of MS due to the dearth of other available effective treatments. Ocrelizumab has been available at AKUHN since 2019.

The purpose of this study was to evaluate the use, safety, and efficacy of anti-CD 20 therapies in patients with MS. The study also served as a structured audit of the clinic’s care practices, providing an evaluation of the consistency of care provided and adherence to established clinical guidelines.

## Materials and methods

### Study design and population

This was a single-centre retrospective chart review conducted at Aga Khan University Hospital Nairobi. The institutional ethics review committee approved the study [2024/ISERC-31 (v3)].

### Inclusion criteria

Patients who fulfilled the McDonald 2017 diagnosis criteria for MS and received at least two doses (induction and maintenance) of either rituximab or ocrelizumab between January 2016 and June 2025 were included in the study. The minimum duration of follow-up was ≥12 months.

### Exclusion criteria

Patients were lost to follow-up for more than 1 year.

### Data collection

Data collected included socio-demographic characteristics (age and sex), medical history (initial symptoms, disease duration since onset, MS phenotype, treatment duration, and detailed history of relapse), previous therapies, reasons for switching to anti-CD therapy (rituximab [RTX] or ocrelizumab [OCR]), the date of RTX or OCR start, dosage of RTX or OCR, and adverse events (AEs).

Adverse events recorded since the initiation of anti-CD 20 therapy were obtained from medical records and magnetic resonance imaging (MRI) findings of the brain done at baseline and every year on follow-up.

To assess efficacy, the relapse rate was reviewed from the clinical notes, MRI findings, and Expanded Disability Status Scale (EDSS) scores (where available) documented before and after the initiation of anti-CD 20 therapy were also noted. Disability was defined as the presence of physical impairment or the requirement of an assistive device. Active disease on MRI was defined by the occurrence of contrast-enhancing T1 hyper-intense or new or enlarging T2 hyper-intense lesions. All scans done at our hospital and external facilities were reviewed independently by our neuroradiologist.

Treatment duration was defined as the interval between baseline and the last available neurological follow-up.

The treatment protocol used in our centre was as follows:

- Rituximab: Induction with two doses of 1,000 mg given intravenously (IV) 2 weeks apart. Maintenance dose of 1,000 mg IV every 6 months.
- Ocrelizumab: Induction with two doses of 300 mg given IV 2 weeks apart and maintenance dose of 600 mg IV every 6 months.

All patients received premedication with paracetamol 1,000 mg, methylprednisolone 100 mg, and chlorpheniramine 10 mg; all were administered intravenously (IV).

### Data analysis

Descriptive statistics on patient characteristics were summarised using mean and standard deviation for continuous variables and percentages for categorical variables. Median and range were provided for abnormally distributed continuous variables. The efficacy of B-cell-targeted therapy was assessed by comparing the rate of disability progression, the number of clinical relapses, and the rate of new/active lesions on MRI and presented in percentages. A *p-*value of ≤0.05 was considered to be significant. All analyses were performed using the Statistical Package for Social Sciences software version 23 (SPSS^©^, Chicago, IL, USA).

## Results

**Figure 1:**
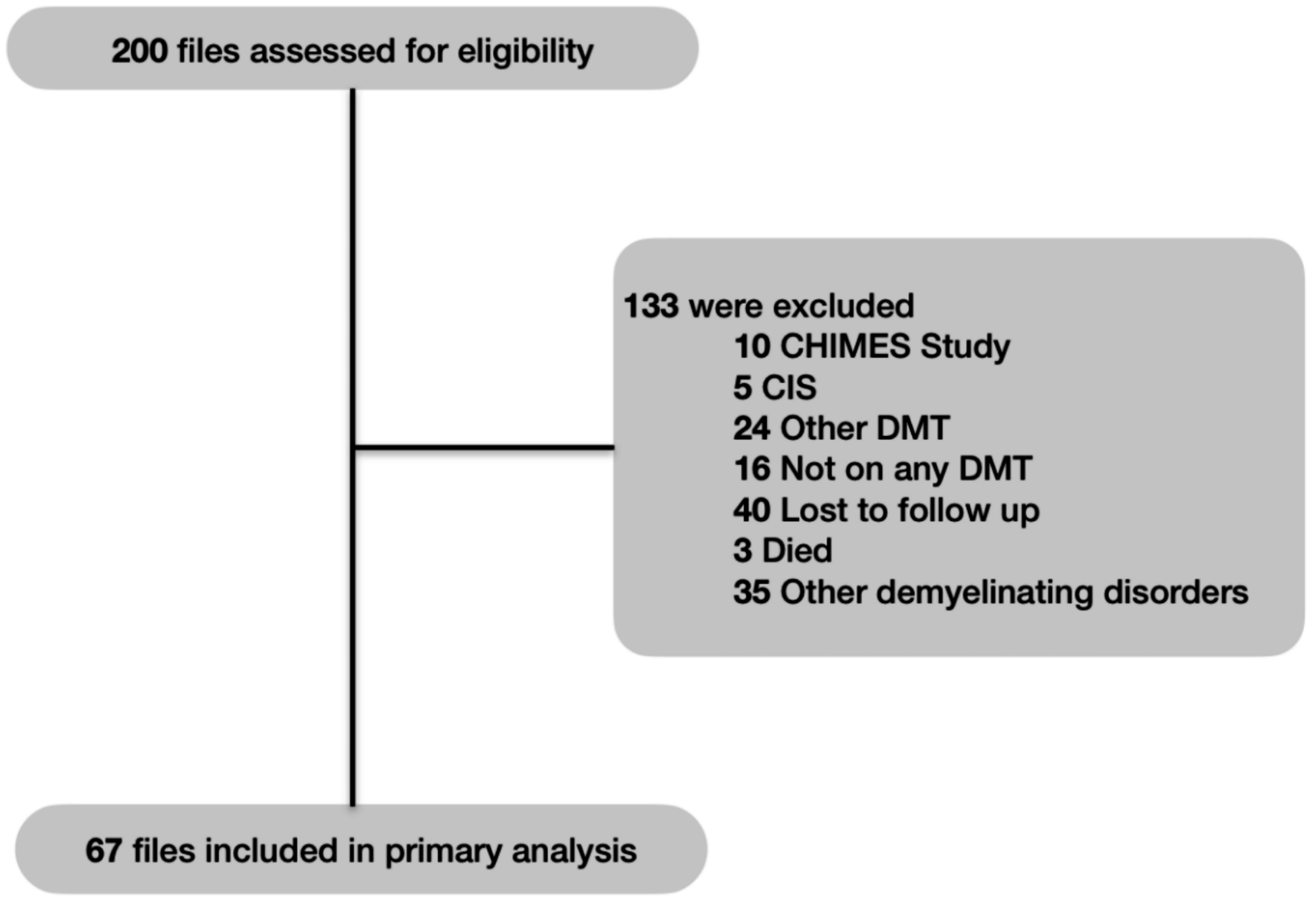
Flow of patient recruitment CHIMES (characterization of ocrelizumab in minorities with multiple sclerosis), CIS (clinically isolated syndrome), DMT (disease-modifying therapy).

A total of 200 files were reviewed, and 133 files were excluded. The 67 patients included in the study were 53 females and 14 males (Figure 1).

The mean age was 40.6 years, with a range of 18–73 years, and the majority were between 18 and 50 years. Amongst all respondents, 39 were married and 66 had attained a tertiary level of education. The median disease duration was 10 years, and the majority (52, 77.6%) had relapsing–remitting MS. The most common symptoms at onset were optic neuritis, sensori-motor manifestations, and ataxia. The socio-demographic characteristics are shown in Table 1.

**Table 1:** Socio-demographic characteristics of the study population.

Variable mean ± SD or *n* (%)	MS patients (*n* = 67)
Age (years), mean (SD)	40.6 ± 13
Mean age at symptom onset (years), mean (SD)	30.3 ± 11.9
Mean age at diagnosis (years), mean (SD)	32.3 ± 11.6
Sex, female, *n* (%)	53 (79.1)
Age	
18–30	16 (23.8%)
31–40	23 (34.3%)
41–50	15 (22.4%)
51–60	7 (10.4%)
> 60	6 (8.9%)
Marital status	
Married	39 (58.2)
Single	25 (37.3)
Separate	1 (1.5)
Divorced	1 (1.5)
Not indicated	1 (1.5)
Level of education, *n* (%)	
Secondary (9–12 years)	1 (1.5)
Tertiary (>12 years)	66 (98.5)
Employment status	
Employed	46 (68.6)
Unemployed	11 (16.4)
Others (retired, not documented)	10 (14.9)
MS phenotype	
RRMS	52 (77.6)
SPMS	10 (14.9)
PPMS	5 (7.5)
Disease duration, n (years)	
<1 year	1 (1.5)
1–5 years	18 (26.8)
≥5 years	48 (71.6)
Mean disease duration, n (years)	10.17 ± 8.4

RRMS, relapsing–remitting multiple sclerosis; SPMS, secondary progressive multiple sclerosis; PPMS: primary progressive multiple sclerosis.

**Figure 2:**
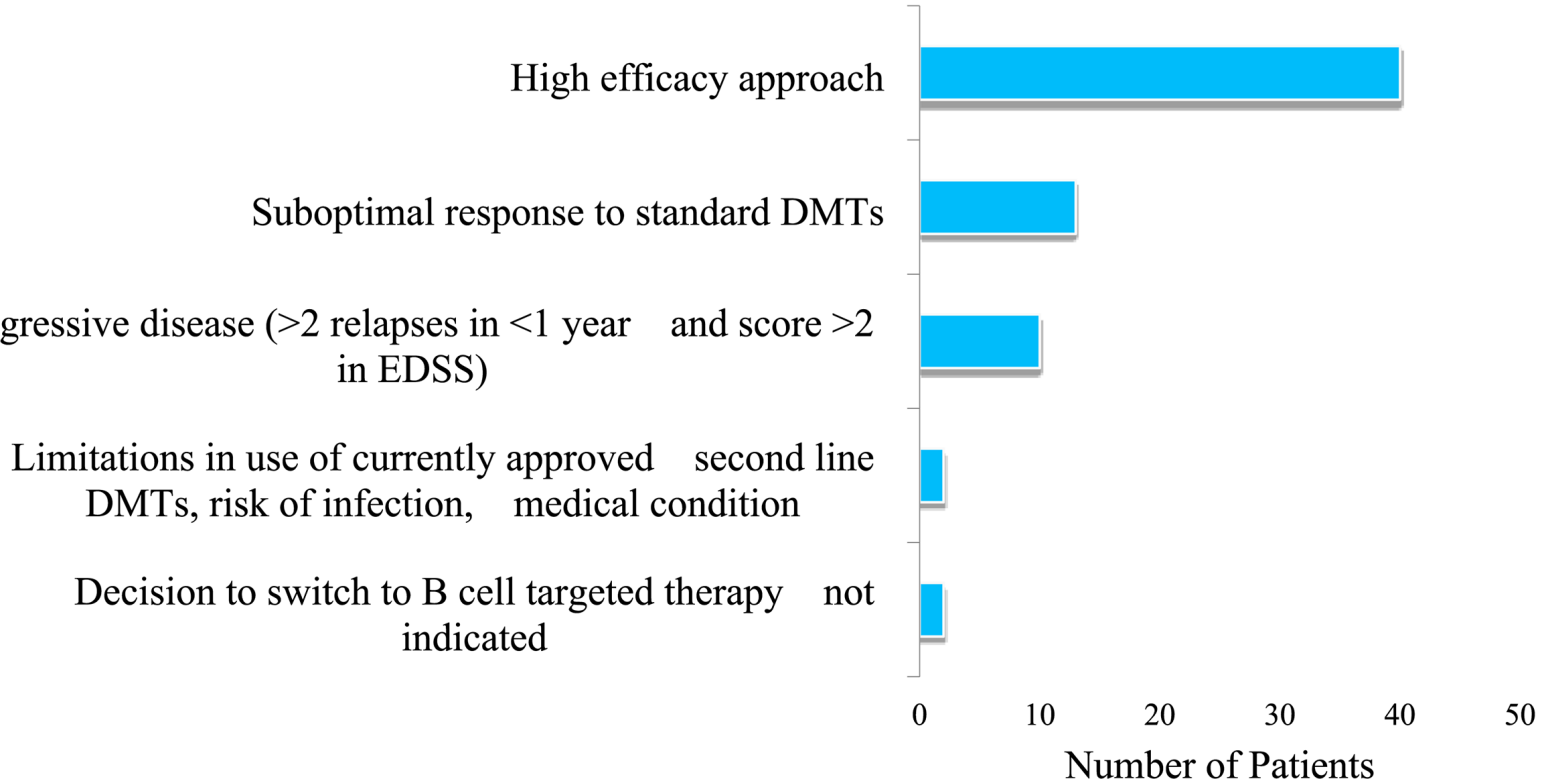
Reasons for initiating rituximab or ocrelizumab.

Over the years, patients have been transitioned to B-cell therapy depending on disease phenotype, patient preference, and other reasons as highlighted below (Figure 2).

**Figure 3:**
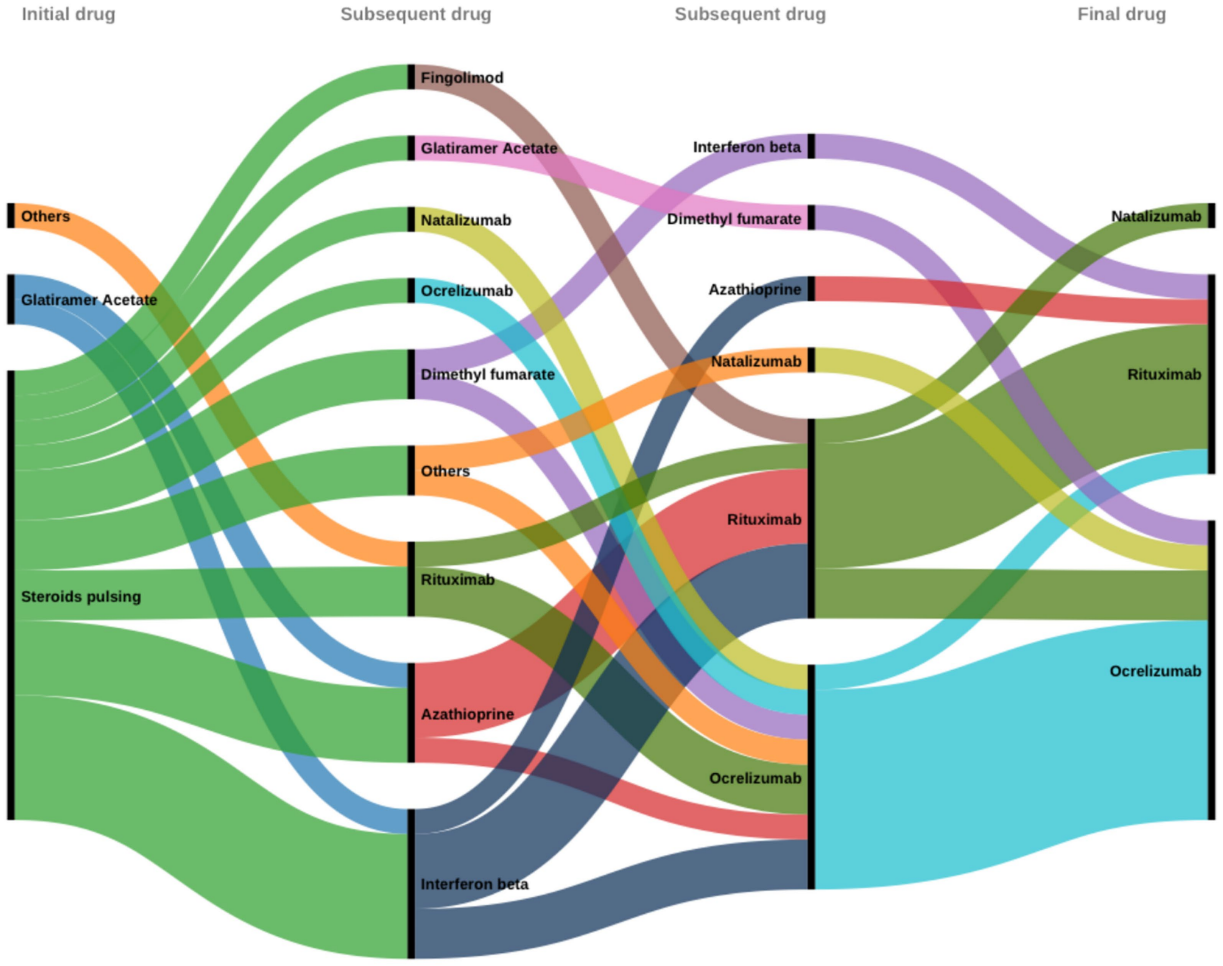
Flowchart showing drug swaps before B-cell-targeted therapy.

Overall, 41 patients were treatment naïve, and 26 patients (38.8%) had used at least 1 or 2 disease-modifying therapies before transitioning to B-cell therapy. Some patients who initially received rituximab were switched to ocrelizumab due to persistent active and new lesions and clinical relapse. Conversely, other patients switched from ocrelizumab to rituximab due to the lower cost of the drug (Figure 3). The average duration since initiation of any B-cell therapy was 4 years. Currently, there are 34 patients on rituximab, 32 patients (excluding 10 patients on the CHIMES trial) on ocrelizumab, and one on natalizumab.

**Figure 4:**
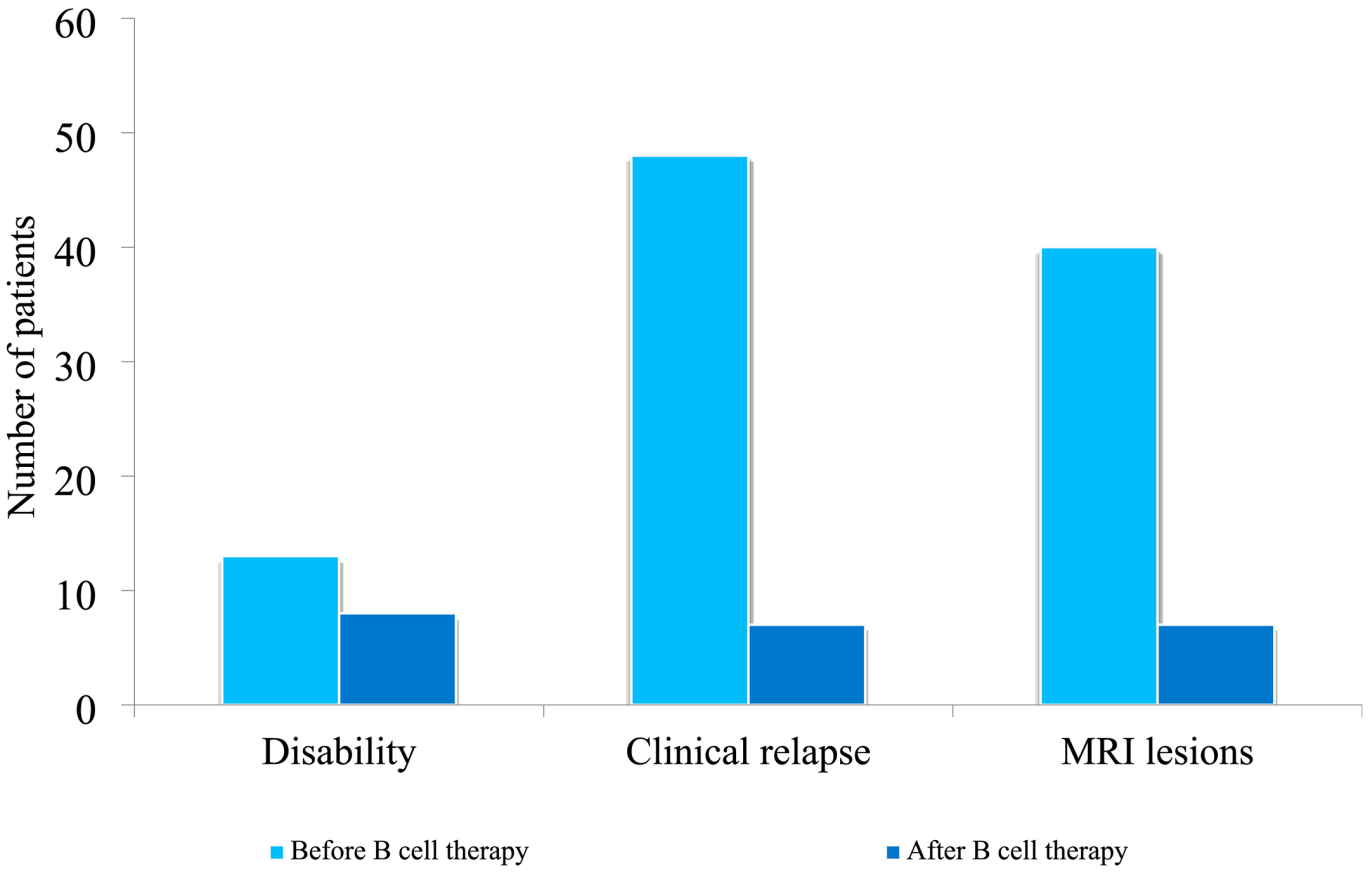
Changes in outcomes before and after B-cell therapy: disability was defined by ambulation status; EDSS was not routinely available.

Thirteen patients had some form of physical disability or were using an assistive device before initiation of any B-cell-targeted therapy. EDSS scores were not used to assess disability, as these scores were only documented for 15 patients, and this was not done consistently either prior to or after treatment. Functional mobility outcome was determined by the proportion of patients who were able to ambulate independently at 1 year following the initiation of B-cell therapy. All patients had a baseline MRI done prior to the initiation of treatment, and on follow-up, 20 (29.8%) did not have a repeat scan after 1 year of treatment. 40 (59.7%) had stable findings, while 7 had active and new lesions. The number of patients having clinical relapse events reduced from 48 to 7. Notable reductions were observed across all parameters (Figure 4).

Overall, there were only four patients who developed upper respiratory tract symptoms, with three having mild symptoms and one mortality due to COVID infection. No infusion-related adverse events were reported. Patients were all screened for tuberculosis and given appropriate immunisations. A total of 7 (10.4%) patients discontinued B-cell therapy, with 4 with progressive disease opting to consider human autologous stem cell transplant and 3 discontinuing due to age and increased risk of infection due to disability.

## Discussion

This study reports the experience of using B-cell-targeted therapy in MS patients in Kenya. The anti-CD 20 therapies used were rituximab (RTX) and ocrelizumab (OCR). A previous study of MS patients in Kenya revealed significant challenges with adherence to disease-modifying therapy. This study also highlighted the limited disease-modifying treatments available and hence the increasing need for more convenient therapeutic options (7). There is a separate ongoing study to assess ocrelizumab (CHIMES) (8), and the patients in that study were excluded from this analysis.

The socio-demographic details are similar to what has been observed previously, with women being predominantly affected. The mean duration of symptoms before official diagnosis also points to challenges in diagnosis and the lag period before referral to a neurologist.

Rituximab binding to CD 20 causes depletion of CD 20-expressing B cells and T cells. Depletion of autoreactive B cells will interfere with cell-mediated antigen presentation and impair T-cell activation, antibody production, and cytokine secretion. Rituximab works through three main mechanisms: induction of apoptosis, complement-dependent cytotoxicity, and antibody-dependent cellular cytotoxicity. Rituximab is widely used in clinical practice but is not approved by the FDA for RRMS. Efficacy in MS was demonstrated in various clinical trials (5). These studies show a notable reduction of disease activity with a reduced annualised relapse rate and formation of T2 gadolinium-enhancing lesions.

Ocrelizumab is a humanised glycosylated anti-CD 20 IgG1 monoclonal antibody that binds to a different but overlapping epitope like that of rituximab. It attaches to the extracellular loop of CD 20 and binds to amino acid residues 165–180. Ocrelizumab was designed to reduce immunogenicity. It selectively depletes CD 20-expressing B cells while preserving capacity for B-cell reconstitution and pre-existing humoral immunity (5). Ocrelizumab has proven efficacy in reducing disability progression and relapse rates in RRMS and PPMS (9).

The use of B-cell-targeted therapy offers a very attractive option with regard to dosing intervals and efficacy (10, 11). The reduction in the number of clinical relapses and the rate of formation of new/active lesions on MRI, as seen in our study patients, shows that these therapies provide incremental benefits to patients (9, 12, 13). One-third of the patients did not have a follow-up MRI at 1 year. Patients have to pay out of pocket for their medical expenses. Lack of an effective publicly funded health system leads to increased cost of care and limits further investigations. We did not analyse the difference in results between rituximab and ocrelizumab. Patients switched between the two drugs for various reasons. Patients who had relapses on rituximab were switched to ocrelizumab, and some patients who have been on ocrelizumab have switched to rituximab due to cost. Given these limitations, it is not easy to make an objective comparison retrospectively, but we are reviewing prospectively the patients who have been on monotherapy.

We also note that patients with very young onset tended to have a more cumulative disability early in their disease process. While an extended dosing interval is an appealing option, patient selection needs to be done judiciously. There were four patients who developed new lesions after defaulting on their biannual treatment. Three patients had clinical relapse and new MRI lesions despite having been on either ocrelizumab or rituximab. This suggests ongoing pathological mechanisms unrelated to B-cell-driven autoimmunity (14).

Given the low side effect profile, these monoclonal antibodies appear to be safe and well-tolerated within our patient population (15). Financial constraints have limited access to B-cell therapy for patients who have no insurance coverage.

## Limitations

Despite the single-centre retrospective design, this study provides important regional data on the safety and efficacy of ocrelizumab and rituximab in MS from an underrepresented population. There was a lack of a control group to assess whether the improved clinical activity was due to the effect of B-cell-targeted therapy or the natural history of the disease. Assessment of disability was limited due to underutilisation of EDSS; this tool has not been adopted for routine clinical use. This was a time-limited study; hence, it was difficult to ascertain the effect of anti-CD 20 therapy on long-term progression of MS disability. Brain and spine MRIs for some patients were done at external facilities, and some imaging centres did not follow the standard protocol for assessing MS. MRI reports were not routinely reported with regard to lesion counts, changes in lesion volumes, and volume/area change of the whole brain and spine. No direct comparisons were made between rituximab and ocrelizumab, as these drugs have been used interchangeably due to various factors.

## Conclusion

This is the largest cohort of patients with MS in Kenya treated with anti-CD 20 therapies. Despite being limited by its single-centre study design, the study highlights treatment outcomes from an African cohort. Early onset portends a more severe disease course. Rituximab and ocrelizumab appear to be safe, well-tolerated, and effective therapeutic options for people with MS. There is a need to effect the World Health Organization essential medicines list 2023 to improve access to DMTs such as anti-CD20 therapies.

## Data Availability

The raw data supporting the conclusions of this article will be made available by the authors, without undue reservation.
